# Status and trends of TMS research in depressive disorder: a bibliometric and visual analysis

**DOI:** 10.3389/fpsyt.2024.1432792

**Published:** 2024-08-08

**Authors:** Jun Yang, Tingting Tang, Qianqian Gui, Kun Zhang, Aixia Zhang, Ting Wang, Chunxia Yang, Xiaodong Liu, Ning Sun

**Affiliations:** ^1^ Department of Psychiatry, First Hospital of Shanxi Medical University, Taiyuan, China; ^2^ First Clinical Medical College, Shanxi Medical University, Taiyuan, China; ^3^ Academy of Medical Sciences, Shanxi Medical University, Taiyuan, China; ^4^ Department of Neurosurgery, First Hospital of Shanxi Medical University, Taiyuan, China

**Keywords:** transcranial magnetic stimulation, depressive disorder, brain mechanisms, efficacy, bibliometrics, visualization analysis

## Abstract

**Background:**

Depression is a chronic psychiatric condition that places significant burdens on individuals, families, and societies. The rapid evolution of non-invasive brain stimulation techniques has facilitated the extensive clinical use of Transcranial Magnetic Stimulation (TMS) for depression treatment. In light of the substantial recent increase in related research, this study aims to employ bibliometric methods to systematically review the global research status and trends of TMS in depression, providing a reference and guiding future studies in this field.

**Methods:**

We retrieved literature on TMS and depression published between 1999 and 2023 from the Science Citation Index Expanded (SCIE) and Social Science Citation Index (SSCI) databases within the Web of Science Core Collection (WoSCC). Bibliometric analysis was performed using VOSviewer and CiteSpace software to analyze data on countries, institutions, authors, journals, keywords, citations, and to generate visual maps.

**Results:**

A total of 5,046 publications were extracted covering the period from 1999 to 2023 in the field of TMS and depression. The publication output exhibited an overall exponential growth trend. These articles were published across 804 different journals, *BRAIN STIMULATION* is the platform that receives the most articles in this area. The literature involved contributions from over 16,000 authors affiliated with 4,573 institutions across 77 countries. The United States contributed the largest number of publications, with the University of Toronto and Daskalakis ZJ leading as the most prolific institution and author, respectively. Keywords such as “Default Mode Network,” “Functional Connectivity,” and “Theta Burst” have recently garnered significant attention. Research in this field primarily focuses on TMS stimulation patterns, their therapeutic efficacy and safety, brain region and network mechanisms under combined brain imaging technologies, and the modulation effects of TMS on brain-derived neurotrophic factor (BDNF) and neurotransmitter levels.

**Conclusion:**

In recent years, TMS therapy has demonstrated extensive potential applications and significant implications for the treatment of depression. Research in the field of TMS for depression has achieved notable progress. Particularly, the development of novel TMS stimulation patterns and the integration of TMS therapy with multimodal techniques and machine learning algorithms for precision treatment and investigation of brain network mechanisms have emerged as current research hotspots.

## 1 Introduction

Depressive disorder is characterized primarily by persistent low mood and cognitive dysfunction. According to a 2019 report by WHO, the global prevalence of depressive disorder is 4.4%. This condition not only affects individuals’ mental health but also significantly interferes with their daily activities and social functioning, imposing substantial burdens on individuals, families, and nations (1, 2). Current treatments for depressive disorder primarily include pharmacotherapy and psychotherapy, although their overall efficacy remains limited (3). With the rapid advancement of non-invasive brain stimulation technologies, TMS has emerged as a promising approach in the field of mental health due to its safety, non-invasiveness, minimal side effects, and ease of use. TMS is an FDA-approved safe and effective non-invasive brain stimulation therapy (4). Initially developed as a non-invasive diagnostic tool to assess neural propagation from the primary motor cortex along the cortical spinal tract, spinal roots, and peripheral nerves (5), it has progressively been applied to treat psychiatric disorders, particularly depressive disorder. Extensive research indicates that TMS demonstrates superior antidepressant effects compared to sham stimulation in emotional symptoms (6), cognitive function (7), suicidal ideation (8), and other aspects (9).

However, with the rapid growth of TMS research, systematically reviewing and evaluating existing research outcomes to identify research hotspots, gaps, and future trends has become crucial for advancing scientific progress in this field. Bibliometric analysis, integrating quantitative analysis of extensive literature data with visualization techniques, can intuitively depict research dynamics, collaboration networks, knowledge maps, and thereby reveal the developmental context and innovative trends within a research domain (10, 11). In the field of depression, some studies have utilized bibliometric techniques, focusing primarily on comorbidities of depression and intervention methods (12–14). Some scholars have also conducted bibliometric analyses on TMS-related research, but these studies tend to concentrate more on other diseases, such as pain, stroke, and Parkinson’s disease (15–17). Therefore, there remains a gap in bibliometric research concerning TMS and depression. This study aims to comprehensively review and deeply analyze recent research outcomes of TMS in the domain of depression, using bibliometric and visualization analysis methods. It systematically reviews the current status of TMS in the treatment of depression, with the objective of guiding future research directions and promoting the scientific application and innovative development of TMS technology in antidepressant therapy.

## 2 Methods

### 2.1 Data sources

**Figure 1:**
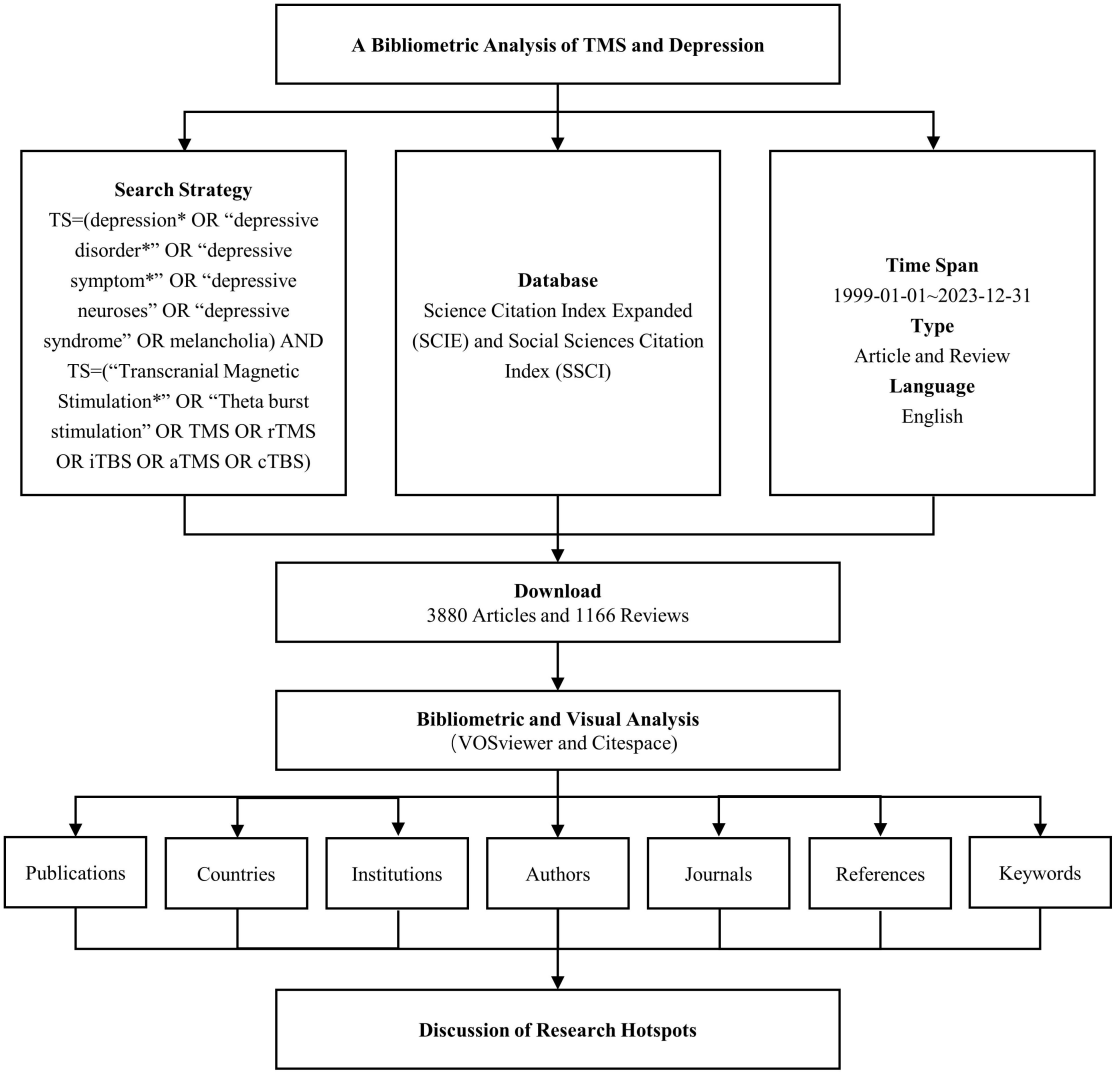
Data retrieval and analysis process. * denotes a truncation character.

Web of Science (WoS) is among the most widely utilized databases for bibliometric analyses. For this study, the Science Citation Index Expanded (SCIE) and Social Sciences Citation Index (SSCI) were chosen from the Web of Science Core Collection (WoSCC) as the databases for the search. The search strategy combined the following terms: TS = (“depression*” OR “depressive disorder*” OR “depressive symptom*” OR “depressive neuroses” OR “depressive syndrome” OR “melancholia”) AND TS = (“Transcranial Magnetic Stimulation*” OR “Theta burst stimulation” OR TMS OR rTMS OR iTBS OR aTMS OR cTBS). The time span for the search was set from January 1, 1999, through December 31, 2023. The search was limited to documents in English and included Article and Review as document types. Following the screening process, a total of 5,046 relevant records were identified, from which data such as publication counts, country affiliations, institutions, authors, journals, keywords, and citation information were extracted. The workflow for data collection is illustrated in **Figure 1**.

### 2.2 Research tool

VOSviewer is a visualization software tool introduced in 2009 by Van Eck and Waltman of Leiden University in the Netherlands. It employs a distance-based visualization methodology (18). In our analysis, VOSviewer (V1.6.19) was employed to examine collaboration networks involving countries, institutions, and authors, along with the keyword density. The specific parameters configured in VOSviewer were as follows: counting method (Full Counting), minimum number of publications for institutions (25), minimum number of publications for authors (20), and minimum frequency of keywords (20).

CiteSpace was created in 2004 by Dr. Chaomei Chen and his team at Drexel University in Philadelphia, USA, using a time and graph-based visualization method (19). We used CiteSpace (V.6.3.R1) to conduct keyword clustering analysis and burst detection. The parameter settings for the CiteSpace software are as follows: time slicing (1 year), K=25, network pruning method (Pathfinder), and the LLR clustering algorithm was used for keyword clustering.

## 3 Results

### 3.1 Annual number of publications

**Figure 2:**
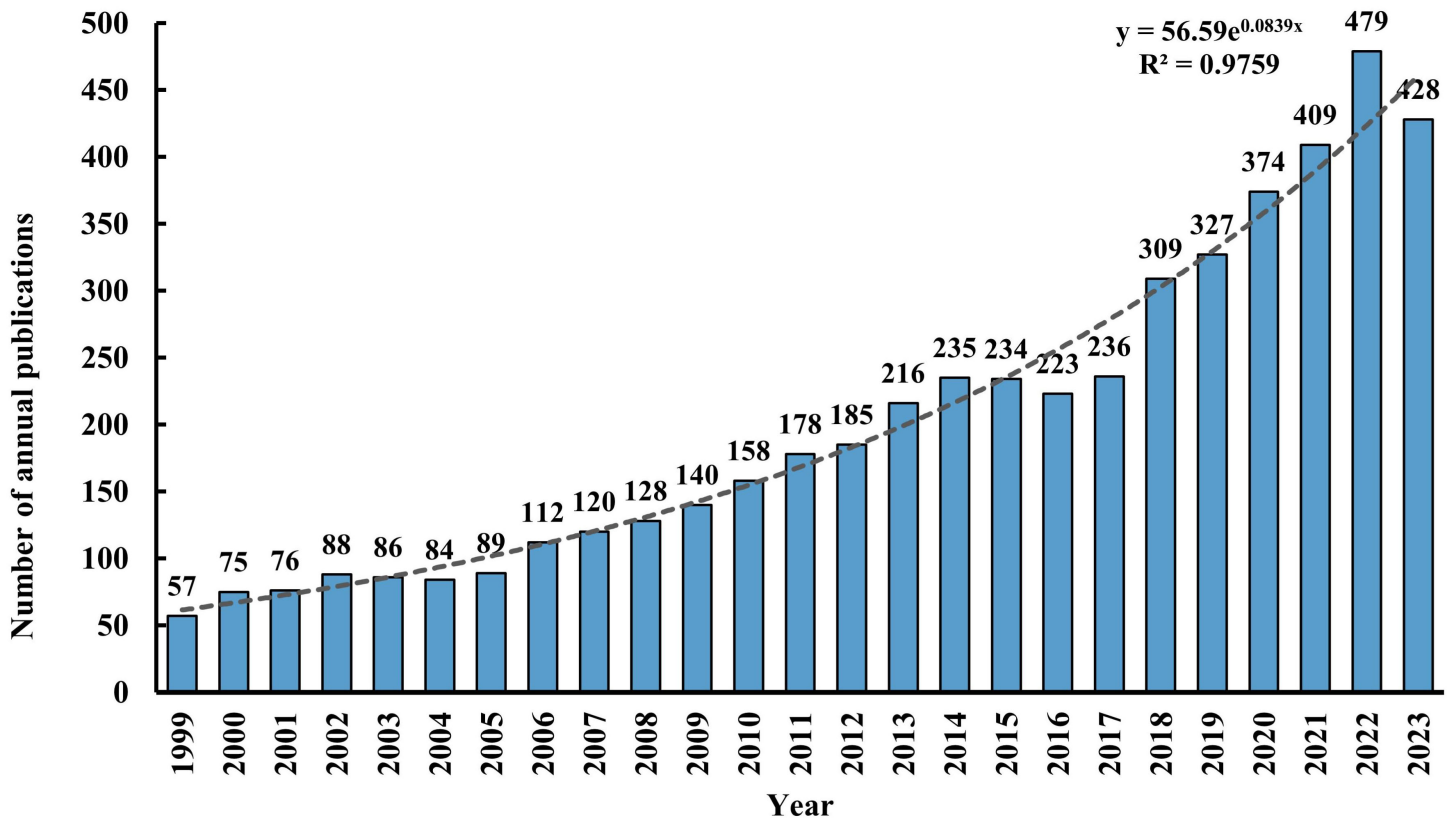
Number of annual publications from 1999 to 2023.

A total of 5,046 records were retrieved from the WOSCC database, comprising 3,880 (77%) articles and 1,166 (23%) reviews. **Figure 2** illustrates the annual publication volume and trend associated with TMS and depressive disorders from 1999 through 2023. The publication count escalated from 57 in 1999 to 428 in 2023, demonstrating an overall exponential growth pattern (R² = 0.9759). It is predicted that the research literature in this field will continue to increase in the future.

### 3.2 Countries and institutions

**Figure 3:**
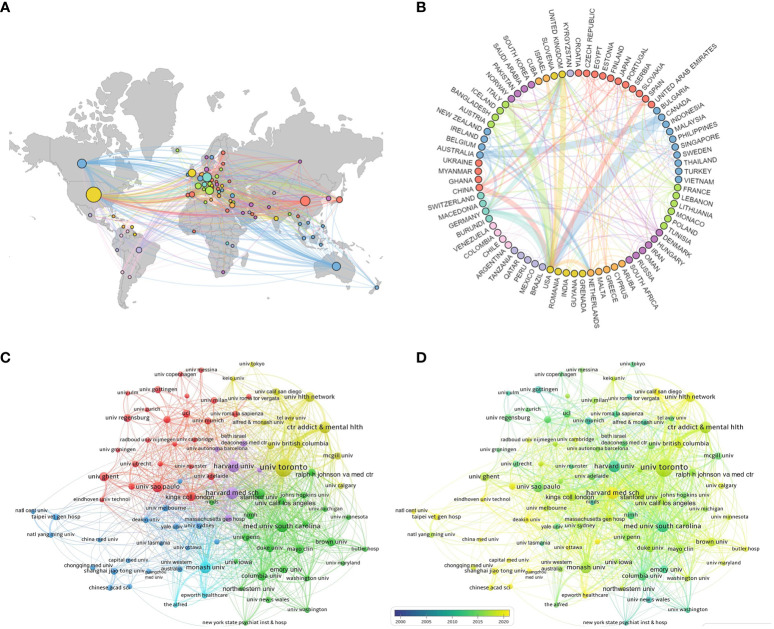
National and institutional collaboration map. **(A)** Geography map of national cooperative. **(B)** Country cooperation relationship map. **(C)** Cluster-based institutional collaboration map. **(D)** Time-based institutional collaboration map.

According to statistical data, a total of 77 countries have contributed to the publication of relevant articles, with the top ten countries based on article quantity detailed in **Table 1**. The United States leads in the volume of publications (N=1,750), trailed by China (N=665) and Germany (N=582). **Figures 3A** and **B** visually represent the worldwide geographic dispersion and collaborative patterns of publications in this discipline. A prominent observation is the concentration of articles originating from regions across North America, South America, Europe, East Asia, and Oceania. The breadth of the connecting lines symbolizes the intensity of collaborative efforts, highlighting the United States’ notably extensive collaboration with other regions, especially with Canada, Italy, and Germany. Additionally, the United States, Germany, the United Kingdom, China, Italy, and France exhibit a centrality exceeding 0.1, underscoring their pivotal roles and significant impact within this research domain.

**Table 1:** Top 10 countries/regions and institutions.

Ranking	Country/Region	Publications	Centrality	Institution	Publications
1	USA	1750	0.36	Toronto Univ	311
2	China	665	0.12	Harvard Med Sch	149
3	Germany	582	0.24	South Carolina Med Univ	143
4	Canada	568	0.09	Ctr Addict & Mental Hlth (CAMH)	138
5	Australia	472	0.05	Monash Univ	138
6	Italy	434	0.11	Harvard Univ	137
7	UK	400	0.16	Stanford Univ	116
8	France	217	0.1	Sao Paulo Univ	115
9	Japan	216	0.03	Univ Hlth Network (UHN)	98
10	Netherlands	206	0.09	Columbia Univ	96

A total of 4,573 institutions have published articles in this field, with the top ten contributing institutions listed in **Table 1**. The University of Toronto emerges as the most prolific and influential institution in the domain, having published a total of 311 articles. Trailing closely are Harvard Medical School (N=149) and the Medical University of South Carolina (N=143). **Figure 3C** illustrates the collaborative network among institutions with ≥25 published articles, where nodes of the same color indicate closer connections, resulting in the formation of five collaborative clusters centered around the University of Toronto, Harvard Medical School, the Medical University of South Carolina, Monash University, and Shanghai Jiao Tong University. **Figure 3D** depicts the temporal dynamics of institutional publications, revealing that both Harvard University and the Medical University of South Carolina initiated research in this area at an earlier stage, whereas Harvard Medical School has emerged as a dominant force in recent years.

### 3.3 Authors

**Figure 4:**
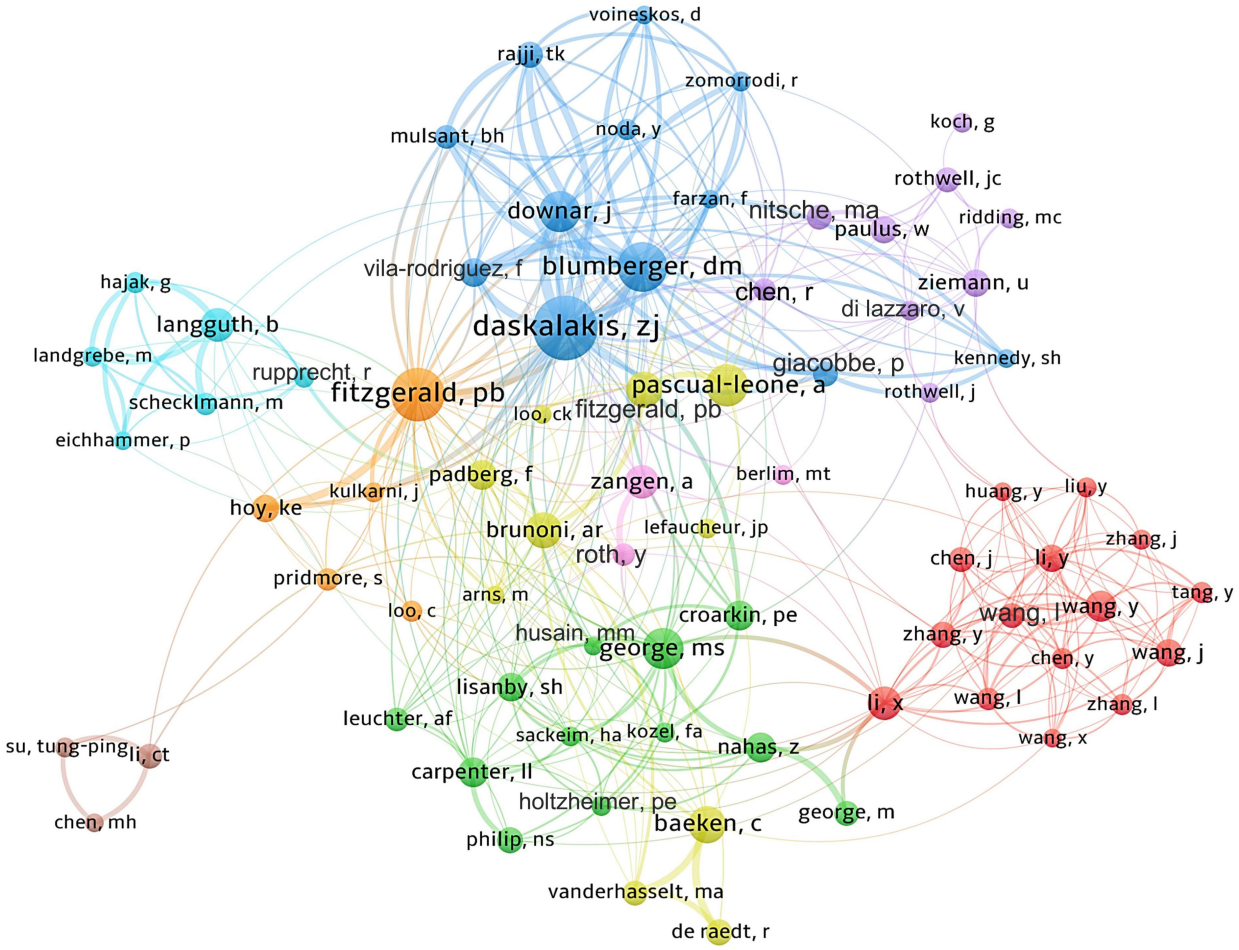
Author collaboration network map.

Approximately 16,000 authors have contributed to the publication landscape in this field. **Table 2** enumerates the top ten authors based on both their publication counts and citation impact. Daskalakis ZJ leads in terms of the number of articles published, with a total of 194 papers, followed by Fitzgerald PB (N=141) and Blumberger DM (N=122). In terms of citation frequency, the author with the highest cumulative citations is Pascual-Leone A (12,677 citations), succeeded by Daskalakis ZJ (10,993 citations) and Fitzgerald PB (9,455 citations). **Figure 4** depicts the collaborative network among authors who have published 20 or more articles, revealing the existence of nine distinct clusters within this intricate web of collaborations.

**Table 2:** Top 10 authors and cited authors.

Ranking	Author	Publications	Cited author	Citations
1	Daskalakis ZJ	194	Pascual-leone A	12677
2	Fitzgerald PB	141	Daskalakis ZJ	10993
3	Blumberger DM	122	Fitzgerald PB	9455
4	Pascual-leone A	113	Paulus W	8740
5	George MS	85	George MS	7936
6	Downer J	85	Fregni F	7137
7	Baeken C	77	Nahas Z	6058
8	Brunoni AR	63	Downer J	5820
9	Fregni F	62	Ziemann U	5358
10	Li X	54	Rothwell J	5218

### 3.4 Journals

The 5,046 articles retrieved were published across 804 different journals, with the top 10 journals by publication volume detailed in **Table 3**. According to the Journal Citation Reports (JCR) classification, half of these top 10 journals are situated in the Q1. Leading amongst them is *BRAIN STIMULATION*, which boasts the highest publication count at 214 articles, accompanied by an Impact Factor of 7.7 and is categorized within the Q1 by JCR. Following closely are *JOURNAL OF AFFECTIVE DISORDERS* (N=191, IF=6.6), *CLINICAL NEUROPHYSIOLOGY* (N=119, IF=3.7), and *FRONTIERS IN PSYCHIATRY* (N=96, IF=3.2) in terms of publication frequency, also reflecting their respective Impact Factors.

**Table 3:** Top 10 journals.

Ranking	Journal	Publications	IF (2023)	JCR (2023)
1	BRAIN STIMULATION	214	7.6	Q1
2	JOURNAL OF AFFECTIVE DISORDERS	191	4.9	Q1
3	CLINICAL NEUROPHYSIOLOGY	119	3.7	Q1
4	FRONTIERS IN PSYCHIATRY	118	3.2	Q2
5	JOURNAL OF ECT	92	1.8	Q3
6	PSYCHIATRY RESEARCH	91	4.2	Q1
7	BIOLOGICAL PSYCHIATRY	78	9.6	Q1
8	FRONTIERS IN HUMAN NEUROSCIENCE	70	2.4	Q2
9	JOURNAL OF PSYCHIATRIC RESEARCH	67	3.7	Q4
10	EXPERIMENTAL BRAIN RESEARCH	64	1.7	Q4

### 3.5 Citation analysis

**Table 4** enumerates the top 10 most co-cited references among all the downloaded literature. Topping the list is an article published in 2018 by Blumberger DM et al. (20) in *THE LANCET*, which accumulated the highest co-citation count (347). This study compares the clinical effectiveness, safety, and tolerability of intermittent Theta-Burst Stimulation (iTBS) versus standard 10 Hz repetitive Transcranial Magnetic Stimulation (rTMS) in adults with treatment-resistant depression (TRD). Ranking second and third in co-citation frequency are the clinical guidelines for the application of rTMS, authored by Lefaucheur JP and colleagues (21, 22).

**Table 4:** Top 10 references.

Ranking	Title	Author	Citations
1	Effectiveness of theta burst versus high-frequency repetitive transcranial magnetic stimulation in patients with depression (THREE-D): a randomized non-inferiority trial	Blumberger DM (20)	347
2	Evidence-based guidelines on the therapeutic use of repetitive transcranial magnetic stimulation (rTMS): An update (2014-2018)	Lefaucheur JP (21)	222
3	Evidence-based guidelines on the therapeutic use of repetitive transcranial magnetic stimulation (rTMS)	Lefaucheur JP (22)	195
4	Consensus Recommendations for the Clinical Application of Repetitive Transcranial Magnetic Stimulation (rTMS) in the Treatment of Depression	McClintock SM (23)	167
5	Efficacy and safety of transcranial magnetic stimulation in the acute treatment of major depression: a multisite randomized controlled trial	OReardon JP (24)	165
6	Daily left prefrontal transcranial magnetic stimulation therapy for major depressive disorder: a sham-controlled randomized trial	George MS (25)	161
7	Repetitive Transcranial Magnetic Stimulation for the Acute Treatment of Major Depressive Episodes: A Systematic Review With Network Meta-analysis	Brunoni AR (26)	152
8	Safety, ethical considerations, and application guidelines for the use of transcranial magnetic stimulation in clinical practice and research	Rossi S (27)	146
9	Resting-state connectivity biomarkers define neurophysiological subtypes of depression	Drysdale AT (28)	140
10	Prospective Validation That Subgenual Connectivity Predicts Antidepressant Efficacy of Transcranial Magnetic Stimulation Sites	Weigand A (29)	135

### 3.6 Keyword analysis

**Figure 5:**
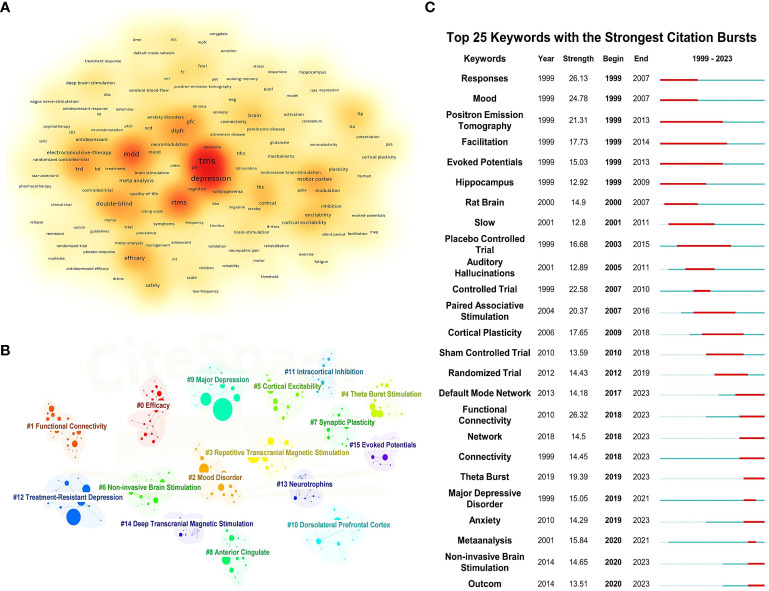
Keyword density map, clustering map, and burst map. **(A)** Keyword density map. **(B)** Clustering map. **(C)** Burst map.

By clustering and density analyzing high-frequency keywords using software algorithms, representative clusters are presented (as shown in **Figures 5A, B**). The application of TMS in the treatment of depressive disorders is reflected in Cluster 2 (Mood Disorder, including depressive disorder, bipolar depressive disorder, dysthymic disorder, etc.), Cluster 9 (Major depression), and Cluster 12 (TRD). As the most common non-invasive physical treatment method, the antidepressant efficacy and stimulation modes of TMS are demonstrated in Cluster 3 (rTMS), Cluster 6 (Non-invasive Brain Stimulation), and Cluster 4 (Theta Burst Stimulation). In terms of micro-level neurobiochemical regulation and macro-level brain region circuitry mechanisms, Cluster 7 (Synaptic Plasticity), Cluster 13 (Neurotrophins), Cluster 8 (Anterior Cingulate), and Cluster 10 (Dorsolateral Prefrontal Cortex) are representative. Cluster 5 (Cortical Excitability) and Cluster 11 (Intracortical Inhibition) can show the excitatory and inhibitory effects of TMS on the brain. Additionally, Cluster 1 (Functional Connectivity) and Cluster 15 (Evoked Potentials) represent the most commonly used brain detection methods for exploring the antidepressant mechanisms of TMS.

**Figure 5C** illustrates the top 25 most cited keywords from 1999 to 2023. The analysis of keyword bursts reflects shifts and trends in the focal points of the research domain, with red segments marking the time of each keyword’s emergence. Keywords that experienced bursts earlier on include “Mood,” “Response,” and “Facilitation,” indicating that initial TMS studies primarily investigated its effects on mood, symptom alleviation, and neural activity in individuals with depressive disorders. As research progressed, terms such as “Controlled Trial,” “Paired Associative Stimulation,” and “Cortical Plasticity” gained prominence, highlighting an evolving interest in the efficacy, safety, and underlying antidepressant mechanisms of TMS among researchers. In recent years, the emergence of keywords like “Default Mode Network,” “Functional Connectivity,” and “Theta Burst” signals the advent of new research hotspots, pointing to the continuous refinement of neuroimaging evaluation metrics and the exploration of novel stimulation paradigms.

**Figure 6:**
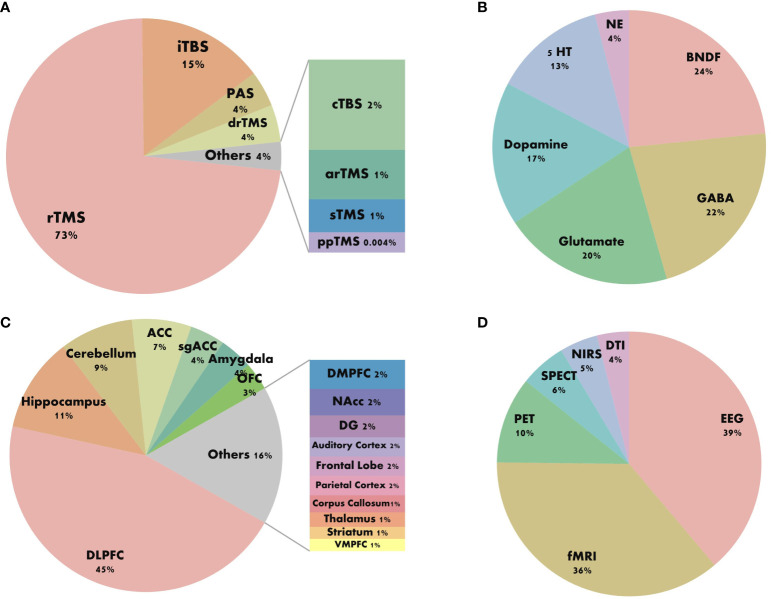
Keyword composition diagram. **(A)** Stimulus pattern. **(B)** Molecular mechanism. **(C)** Brain area. **(D)** Combined techniques.

We standardized the keywords of the articles and categorized the stimulation patterns of TMS, BDNF, neurotransmitters, brain regions, and combined detection technologies in a standardized manner (as shown in **Figure 6**). **Figure 6A** indicates that the most commonly used stimulation pattern for TMS is repetitive rTMS, followed by intermittent theta burst stimulation (iTBS). **Figure 6B** reveals that among the micro-molecular studies of TMS in depression, BDNF is the most frequently explored, followed by gamma-aminobutyric acid (GABA) and glutamate. **Figure 6C** shows that in the research on the stimulation targets and brain regions of TMS in depression, the dorsolateral prefrontal cortex (DLPFC) is the most studied, followed by the hippocampus and cerebellum. The anterior cingulate cortex (ACC) and subgenual anterior cingulate cortex (sgACC) have also been extensively investigated. From **Figure 6D**, we can observe that the research on the mechanism of TMS efficacy is mostly based on Electroencephalogram (EEG) and Functional Magnetic Resonance Imaging (fMRI) technologies.

## 4 Discussion

What is the current global research landscape concerning TMS for treating depression? What significant achievements and research trends have emerged in the treatment and mechanistic study of depression? Our visual analysis and systematic review offer a comprehensive overview of the current research status in this field.

### 4.1 The global research trends of transcranial magnetic stimulation in the field of depression disorders

From 1999 to 2023, there has been a sustained increase in research on TMS and depressive disorder, reflecting rapid advancements in both scientific research and clinical applications in this field. Among the numerous journals publishing research outcomes related to TMS and depression, *BRAIN STIMULATION*, a leading international authority in the neurostimulation domain, has distinguished itself through its exceptional academic publishing standards and widespread scholarly influence. It has disseminated numerous high-quality research papers, establishing itself as the most prolific journal in this field in terms of publications.

This study encompasses 4,573 institutions across 77 countries, illustrating global attention and participation in TMS research for depression, particularly concentrated in North America, Europe, East Asia, and Oceania. The United States leads significantly with 1,750 publications, showcasing its robust strength and extensive expertise in TMS and depression research. This leadership is evident not only in quantity but also in extensive international collaborations, notably with Canada, Italy, and Germany. China follows with 665 publications, reflecting its rapid emergence and strong potential in this field, supported by increased research investment and improved scientific environments, positioning China as a significant future force. Prominent institutions such as the University of Toronto, followed closely by Harvard Medical School and the Medical University of South Carolina, play pivotal roles as influential hubs in this field. These institutions not only demonstrate high productivity but also lead in network collaboration and research direction. Early contributions from Harvard University and the Medical University of South Carolina have laid foundational groundwork, while recent advancements from Harvard Medical School underscore ongoing and dynamic developments in this field. Notably, 8 of the top 10 ranked institutions are globally renowned universities, reflecting dominance in research driven by high-caliber platforms and talent. Furthermore, half of these top-ranked institutions originate from the United States, reaffirming its leadership and pivotal role facilitated by substantial research investments, advanced facilities, and a concentration of top academic institutions. Among over 16,000 authors, figures like Daskalakis ZJ, Fitzgerald PB, and Pascual-Leone A not only publish prolifically but also wield substantial academic influence, through close collaboration with other authors, they have gradually formed their own circles of cooperation. The identification of 9 clusters likely represents distinct research teams, indicating a trend towards enhanced collaboration and expanded cooperative networks over time. In summary, with ongoing technological advancements and deepened research endeavors, TMS holds promising prospects for depression treatment. Future expectations include the emergence of higher-quality research outcomes and broader international and interdisciplinary collaborations.

### 4.2 The status and hotspots of transcranial magnetic stimulation in the field of depression disorders

#### 4.2.1 TMS stimulation modes, efficacy, and safety

TMS is a non-invasive neuro-modulation technique categorized into three main modes based on pulse stimulation: single-pulse TMS (sTMS), paired-pulse TMS (ppTMS), and rTMS. Initially employed in studying brain function and assessing neurological disorders, TMS has evolved into a crucial therapeutic tool in psychiatric illnesses. ppTMS involves delivering two stimuli to the same target area using a single coil or stimulating different regions with two coils, commonly utilized to investigate neural facilitation and inhibition mechanisms. Jeng et al. observed significant reductions in short-interval intracortical inhibition (SICI) and long-interval intracortical inhibition (LICI) in TRD patients using ppTMS (30). When the paired stimuli in ppTMS target different cortical and peripheral neural regions respectively, this mode is known as Paired Associative Stimulation (PAS). Noda et al. demonstrated impaired neuroplasticity in the DLPFC of depressive patients using the DLPFC-PAS paradigm, characterized by reduced power in gamma (γ), theta (θ), and delta (δ) frequency bands, and diminished phase-amplitude coupling (θ-γ coupling) (31).

rTMS is widely approved as a first-line treatment for depressive disorder and represents the most utilized modality for this condition (**Figure 6A**). By delivering either continuous or intermittent stimulation to specific brain regions, rTMS induces lasting neuroregulatory effects. The predominant stimulation protocols for treating depression involve high-frequency stimulation (HF) to the left-DLPFC(L-DLPFC) and low-frequency stimulation (LF) to the right-DLPFC(R-DLPFC) (26). HF-rTMS, defined as stimulation exceeding 1 Hz, exerts excitatory effects on cortical activity. Meta-analyses indicate that optimal antidepressant effects are achieved when HF-rTMS sessions deliver a total pulse count between 1200 and 1500 pulses per day (9). LF-rTMS, characterized by stimulation at 1 Hz or less, conversely inhibits cortical activity. A multicenter randomized double-blind controlled trial has demonstrated that continuous 1 Hz LF-rTMS administered over 2-6 weeks produces significant therapeutic benefits in TRD, with a response rate of 41% (32).

With the advancement of TMS technology, several new stimulation modes have emerged such as Theta Burst Stimulation (TBS), accelerated rTMS (arTMS), and deep rTMS (drTMS). TBS, by mimicking the endogenous theta rhythm of the hippocampus in pulse release frequency (33), significantly induces synaptic long-term potentiation. Among these, iTBS has received FDA approval for treating treatment-resistant depression, offering advantages of shorter stimulation time and stronger induction of neuroplasticity compared to conventional rTMS. Derivative modes such as accelerated iTBS (aiTBS) and prolonged iTBS (piTBS) are increasingly recognized in clinical studies for depression treatment (34). A randomized controlled trial from Canada initially compared iTBS with 10 Hz rTMS, demonstrating comparable efficacy in reducing depressive symptoms, with response and remission rates of 49% and 32%, respectively, for the 3-minute iTBS protocol versus the standard 37.5-minute 10 Hz rTMS protocol (20). Kishi et al. reviewed six randomized controlled trials supporting similar findings and suggesting that iTBS, with its shorter stimulation time and lower intensity, may be more suitable in clinical applications than HF-rTMS (35). In contrast, continuous TBS (cTBS) reduces neuronal excitability, and a combined approach of cTBS over the right DLPFC with iTBS over the left DLPFC demonstrates effective antidepressant effects (22). arTMS, characterized by shorter duration and higher frequency of stimulation, proves faster and more effective in improving major depressive episode compared to standard rTMS protocols (36). Utilizing an H-coil system, drTMS allows stimulation of deeper brain regions. Levkovitz et al. demonstrated the efficacy and safety of 18 Hz drTMS in 212 major depressive disorder (MDD) patients resistant to antidepressant medication, maintaining therapeutic effects for up to 16 weeks (37).

In recent years, the efficacy and safety of TMS in MDD have been extensively researched. A meta-analysis involving 2,982 depressed patients demonstrated significant improvement in depressive symptoms following rTMS treatment compared to sham stimulation (Hedges’g = -0.791), with more than twice the likelihood of response or remission in the rTMS group (38). Li et al. demonstrated the efficacy of various TMS protocols for treating TRD and TBS to potentially be the most effective mode (39). Qiu et al.’s study validated the effectiveness of TMS in treating adolescent depression, showing comparable response rates between adolescents (33%-56%) and adults (29%-60%), and even higher remission rates in adolescents (13%-44%) compared to adults (18%-22%) (40). Similarly, rTMS effectively alleviates depressive symptoms in elderly patients, with treatment outcomes improving with advancing age (41). Wang et al. also confirmed the safety and tolerability of TMS for MDD treatment, noting minor and transient adverse effects such as discomfort and pain at the stimulation site (42).

#### 4.2.2 Brain region mechanisms based on combined techniques

The application and advancement of brain imaging techniques in conjunction with TMS, such as EEG and fMRI, have become prominent. These techniques assist in neuro-navigation for targeting stimulation areas, exploring underlying psychophysiological mechanisms, and predicting individualized therapeutic efficacy. EEG, with its high temporal resolution, is a common tool in TMS research. In recent years, the maturity of TMS-EEG technology has enabled simultaneous stimulation of brain tissues and real-time acquisition of electroencephalographic signals, facilitating immediate monitoring of stimulation effects. Furthermore, fMRI, renowned for its exceptional spatial resolution, reveals subtle changes in brain functional connectivity under depressive disorders, offering valuable insights into the neurobiological basis of depression (43, 44).

In numerous TMS studies, the DLPFC has held a pivotal position as a core target for treating depressive disorders (**Figure 6C**). Researchers associate the DLPFC primarily with functions related to emotion regulation, reward processing, cognition, and decision-making assessment, all of which are impaired in depressive disorders, closely linking its diagnosis and treatment (45). Situated in the superficial cortex of the brain, the DLPFC can be directly stimulated via TMS pulses through the skull, underscoring why it has long been considered a classical stimulation target (46). However, as research advances, scientists are increasingly exploring other brain regions such as the hippocampus, cerebellum, ACC, amygdala, and orbitofrontal cortex (OFC), all of which play complex and crucial roles in the neurophysiological mechanisms of depression.

With the rise of MRI-based studies on brain connectivity and the introduction of brain network concepts, it is increasingly acknowledged that TMS stimulation effects can propagate to distant brain regions through network effects, potentially modulating entire basal brain networks. Many scholars attribute the therapeutic efficacy of rTMS in treating depression largely to stimulation effects spreading through functional connections from the stimulated area to deeper brain structures (47). The hippocampus is recognized as a crucial brain region involved in learning, memory, and co-regulation of perception and emotion with other brain areas (48). Structural and functional abnormalities in the hippocampus are closely linked to psychiatric disorders, particularly MDD (49, 50). A study analyzing TMS-EEG data in MDD patients before and after rTMS treatment found that the orbitofrontal-hippocampal pathway plays a pivotal role in mediating depression relief post rTMS therapy, suggesting potential alternative targets for brain stimulation therapies targeting depression (51).

Traditionally regarded as part of the motor circuitry, the cerebellum’s role in cognitive functions is increasingly recognized. Cerebellar outputs can project directly to the cerebral cortex, modulating excitability through tightly integrated neural circuits with the neocortex. Meta-analyses based on neuroimaging studies indicate that, apart from the frontal lobes, both structural and functional alterations in the cerebellum and brainstem are implicated in the pathophysiology of TRD (52). Recent research suggests that the cerebellum could serve as an alternative target for rTMS therapy in depression patients. Concurrent stimulation of the prefrontal cortex and cerebellum may potentially enhance symptom improvement more effectively in these patients, with ongoing efforts aimed at experimental validation. A PET study revealed that TRD patients who exhibited better clinical outcomes and responded to left DLPFC HF-rTMS treatment showed higher baseline cerebellar metabolic activity (53).

Research indicates that the ACC is implicated in emotion regulation and modulates reward and non-reward mechanisms in depression (54). The rostral anterior cingulate cortex (rACC), a critical component of the ACC located anterior to the corpus callosum, serves as a hub within the default mode network (55). In a multicenter randomized clinical trial, increased theta activity in the rACC predicted improvement in depressive symptoms among MDD patients, suggesting it as a nonspecific prognostic biomarker for treatment outcomes (56). The sgACC, another integral part of the ACC, is closely associated with depression, particularly in the regulation of sadness (57). Previous studies have shown that better outcomes are associated with transference of stimulation pulses from the DLPFC to regions negatively connected with the sgACC (29, 58). Ge et al.’s rTMS-fMRI study on TRD indicated that functional connectivity levels between sgACC and rACC may serve as potential predictors of rTMS treatment response, demonstrating robustness in longitudinal follow-ups up to three months (59). Additionally, Baeken et al. used PET imaging to explore metabolic changes in the sgACC post-treatment, observing decreased local glucose metabolism in TRD patients following transcranial magnetic stimulation (60). Abnormal functional connectivity among subregions of the anterior cingulate cortex appears to be crucial in depression circuits and mechanisms underlying rTMS treatment effects, warranting further investigation in ACC-focused research.

The amygdala is a crucial component of reward and salience networks and a key site implicated in chronic stress-induced alterations in patients with depressive disorders (61). Eshel et al., using rTMS-fMRI with the L-DLPF as a stimulation target, demonstrated increased global connectivity in patients receiving active stimulation. This intervention induced restoration of inhibitory connectivity between L-DLPFC and the amygdala, changes in which could predict clinical outcomes (62). Besides the aforementioned brain regions, various subregions of the frontal cortex Dorsomedial Prefrontal Cortex (DMPFC), Ventromedial Prefrontal Cortex (VMPFC), as well as the parietal cortex, auditory cortex, and visual cortex, have garnered increasing attention from researchers. Several of these areas are considered as potential alternative stimulation targets to DLPFC.

The exploration of TMS-induced metabolic, functional, and structural changes in different brain regions among patients with depression has become a recent focus of research. With the rising adoption of multimodal integration technologies, particularly multi-channel and multimodal approaches, robust tools have emerged for real-time monitoring of dynamic brain changes under TMS intervention. These advancements not only facilitate precise spatial localization for targeted TMS therapies but also enable exploration of the macroscopic mechanisms of temporal and spatial information across different brain regions. However, these macroscopic changes in brain connectivity and networks to some extent reflect microscale alterations in synaptic plasticity and neurotransmitter expression. Next, our focus shifts to the micro mechanisms of TMS antidepressant effects, analyzing how they operate at the molecular level.

#### 4.2.3 Molecular mechanism

rTMS induces long-lasting excitatory or inhibitory postsynaptic potentials in neurons by high-frequency repetitive stimulation. This phenomenon, known as long-term potentiation (LTP) or long-term depression (LTD), persists over extended periods. Extensive stimulation can alter the adjustment and reorganization of connections between neurons, influencing the expression of neurotrophic factors and neurotransmitters within the brain. These effects may be linked to alterations in gene expression and protein synthesis at deeper levels. Previous research has indicated that TMS can modulate levels of BDNF and neurotransmitters (GABA, glutamate, DA, 5-TH) in patients with depressive disorders. Changes in these neurotransmitter levels reflect the impact of TMS on the micro-metabolic state of the brain and are closely related to clinical outcomes in patients.

BDNF is associated with MDD and many other neuropsychiatric disorders (63), playing a crucial role in synaptic plasticity (64). A study employing high-dose rTMS guided by neuronavigation in depressed patients demonstrated that responders to rTMS exhibited elevated BDNF levels compared to sham stimulation controls (65). Another controlled study reported increased BDNF levels following rTMS, which inversely correlated with depressive symptom severity (66). Fundamental research suggests that rTMS modulates BDNF levels, thereby exerting neurotrophic and neuroprotective effects. The neuroprotective action of rTMS may involve promoting neuronal proliferation and differentiation while inhibiting apoptosis. Feng et al. found that rTMS treatment over 3 weeks in a depression model of rats promoted cell proliferation in the hippocampal region (67). Moreover, rTMS suppresses apoptosis mainly through modulation of apoptosis-related protein expression levels. Gao et al. applied 20 Hz rTMS for 7 consecutive days in rats with transient cerebral ischemia and observed upregulation of Bcl2 expression along with downregulation of Bax expression (68).

Under normal circumstances, there exists a delicate balance between GABA and Glu in the central nervous system. The inhibitory action of GABA counteracts the excitatory effects of Glu, thereby maintaining normal neural function. Research indicates that rTMS can modulate the synthesis and release of Glu and GABA in the CNS. Using MRS, studies have investigated changes in these neurotransmitters during treatment. One study found that after 20 sessions of 10Hz rTMS, there was no significant change in GABA levels in the left DLPFC, while Glu levels increased (69). Another study using MRS measured GABA levels before and after 25 sessions of 10Hz rTMS, revealing a significant elevation in prefrontal cortex GABA levels post-treatment. These findings are crucial for understanding the neurochemical mechanisms of depression and developing new therapeutic approaches (70). Additionally, research has shown that after iTBS treatment, the GABA/Glx ratio decreased in the frontal cortex, reflecting alterations in the balance between excitatory and inhibitory processes within the brain (71). Basic experimental studies have indicated that TMS stimulation can influence the activity of GABA transporters and the expression of GAD 65/67, leading to increased levels of frontal Glu/Gln and NAA. Furthermore, studies applying rTMS to rats for 15 days demonstrated significant increases in GABA and Glu release in the hippocampus and striatum, while levels in the hypothalamus decreased (72). Trippe et al. observed changes in the expression of glutamate decarboxylase (GAD) following different patterns of rTMS stimulation in rats: upregulation of GAD65 expression and downregulation of GAD67 expression (73).

Neuroimaging studies have shown that TMS can modulate the reward circuitry in patients with depression, thereby exerting antidepressant effects. Researchers propose a mechanistic perspective: TMS enhances dopamine release in brain regions associated with the reward network (e.g., prefrontal cortex, amygdala, striatum, and ventral tegmental area), potentially ameliorating anhedonia and cognitive impairments in individuals with depressive disorders. A PET study revealed a significant increase in dopamine levels following rTMS stimulation of the left prefrontal cortex, suggesting a facilitative effect of rTMS on dopamine release (74). However, another PET study utilizing L-[b-11C] DOPA to assess endogenous dopamine synthesis rates found no change in striatal dopamine synthesis rate (k value) after a 10-day course of rTMS treatment in 8 patients with depressive disorders (75). Controversies and uncertainties persist regarding the precise impact of TMS on dopamine dynamics.

Most antidepressant medications (e.g., SSRIs) exert their effects by modulating the serotonin system, and the antidepressant action of TMS may also involve modulation of 5-HT levels. A sham-controlled rTMS study indicated no change in plasma serotonin levels following rTMS treatment, yet binding of 5-HT2A receptors (a subtype of the serotonin receptor family) in the brain correlates positively with symptom improvement in the DLPFC and negatively in the hippocampus (76). At a microscopic level, TMS affects not just one neurotransmitter but modulates neurotransmitter levels across multiple neural circuits, thereby influencing overall brain excitatory-inhibitory balance and neuroplasticity, ultimately leading to improvements in depressive symptoms. Overall, TMS operates through multilayered, multidimensional regulatory mechanisms to rebalance brain function. The interactions of these mechanisms form a complex regulatory network, necessitating comprehensive exploration of its deeper therapeutic mechanisms.

### 4.3 Limitations

This study utilized bibliometric methods to analyze the evolution and trends in TMS research in depression. Bibliometrics has several inherent limitations (77, 78). Firstly, one limitation is that search terms may be restricted, potentially leading to incomplete retrieval. Nevertheless, we believe that the majority of TMS studies in depression have been included, with a sufficiently large sample size. Secondly, while WoSCC is the most commonly used and authoritative comprehensive database, this study exclusively utilized bibliometric data from WoSCC, overlooking other large databases (79). Thirdly, another limitation of this study is that due to software constraints, only English-language publications were analyzed, thereby overlooking several high-quality non-English articles. Lastly, we did not assess the quality of publications, hence treating high-quality and low-quality publications equally in our analysis.

## 5 Conclusion

This study employed bibliometric methods for the first time to review the research history and current status of TMS in the field of depression from 1999 to 2023. It conducted a visual analysis of publication trends, countries, institutions, authors, journals, and keywords. The study revealed a sharp increase in relevant literature in recent years, highlighting significant attention and rapid development in this area. The United States emerged as the leading contributor to research output, with Toronto University and Daskalakis ZJ prominent in institutional and individual contributions. The journal *BRAIN STIMULATION* published the most articles in this field.

Through an in-depth exploration of stimulation patterns, molecular mechanisms, brain regions, and integration with other brain imaging technologies, this study delineates the research focus and cutting-edge directions of TMS in the treatment of depression. Enhancing the antidepressant efficacy of TMS and probing its underlying brain neurobiological mechanisms have been the focal points of scientific endeavors over the past 25 years. The trend towards personalized precision therapy persists, leveraging individual-specific multimodal parameters, machine learning, and big data analytics. While neuro-navigation techniques aid in achieving precise targeting, initial successes have been observed in rTMS treatments tailored to specific depressive symptoms based on specific neural circuits. Nonetheless, numerous challenges remain, including standardizing TMS stimulation parameters, constructing treatment response prediction models, and conducting long-term follow-up studies to comprehensively assess research status and optimize the therapeutic potential of TMS. Alongside the evolution of multimodal techniques in machine learning, radiomics integrates analysis across multiple dimensions such as patient genotypes, phenotypes, brain structure, and function, facilitating early diagnosis and treatment optimization for depressive disorders. This approach aims to achieve personalized dynamic treatment feedback, thereby amplifying the therapeutic impact of TMS in treating depressive disorders.

In summary, TMS technology demonstrates extensive potential applications and significant implications for treating depressive disorders. The hotspot analysis and trend insights from this study provide valuable direction for future research, offering a solid reference point to potentially advance and innovate depression treatment techniques.

## Data availability statement

The original contributions presented in the study are included in the article/supplementary material. Further inquiries can be directed to the corresponding authors.

## Author contributions

JY: Conceptualization, Formal analysis, Writing – original draft. TT: Data curation, Formal analysis, Writing – original draft. QG: Data curation, Writing – original draft. KZ: Data curation, Writing – original draft. AZ: Conceptualization, Writing – review & editing. TW: Writing – review & editing. CY: Funding acquisition, Writing – review & editing. XL: Writing – review & editing. NS: Funding acquisition, Methodology, Writing – review & editing.
